# Lipid-lowering drug prescriptions in a group of Colombian patients

**DOI:** 10.7705/biomedica.4801

**Published:** 2019-12-30

**Authors:** Andrés Gaviria-Mendoza, Manuel E. Machado-Duque, Jorge E. Machado-Alba

**Affiliations:** 1 Grupo de Investigación en Farmacoepidemiología y Farmacovigilancia, Universidad Tecnológica de Pereira-Audifarma, S. A., Pereira, Colombia Universidad Tecnológica de Pereira Grupo de Investigación en Farmacoepidemiología y Farmacovigilancia Universidad Tecnológica de Pereira Pereira Colombia; 2 Grupo de Investigación en Biomedicina, Fundación Universitaria Autónoma de las Américas, Pereira, Colombia Grupo de Investigación en Biomedicina Fundación Universitaria Autónoma de las Américas Pereira Colombia

**Keywords:** Dyslipidemias, ezetimibe, pharmacoepidemiology, hydroxymethylglutaryl-CoA reductase inhibitors, hypolipidemic agents, drug prescriptions, dislipidemias, ezetimiba, farmacoepidemiología, inhibidores de hidroximetilglutaril-CoA reductasas, hipolipemiantes, prescripciones de medicamentos

## Abstract

**Introduction.:**

Lipid-lowering drugs, especially statins, have shown great relevance in preventing and treating cardiovascular diseases.

**Objective.:**

To determine the prescription patterns of lipid-lowering drugs and the variables associated with their use in a Colombian population.

**Materials and methods.:**

This is a cross-sectional descriptive study. From a drugdispensing database of approximately 4.5 million Colombian health system affiliates, patients of all ages and both sexes treated with lipid-lowering agents (statins, fibrates, ezetimibe) were identified between January and March, 2017. Demographic, pharmacological and co-medication variables were included.

**Results.:**

In total, 103,624 patients were identified as being treated with lipid-lowering agents. The average age was 67.5 years, and 49.8% were 65 years or older. Women comprised 58.0% of the patients. Statins were the most used (n=96,910; 93.5%), and atorvastatin (n=80,812; 78.0%) and lovastatin (n=12,621; 12.2%) were the most frequent. The mean atorvastatin dose was 30.3 mg/day, and 49.9% of its users received presentations of 40 mg or more. A total of 9,258 (8.9%) patients received fibrates, and only 780 (0.8%) were taking ezetimibe. Of this population, 94.9% were treated with lipid-lowering monotherapy, and 97.3% (n=100,813) had co-medication for their comorbidities, with the most frequent being antihypertensive (89.1%), antiplatelet (57.8%), antidiabetic (31.5%) and antiulcerative agents (34.2%).

**Conclusions.:**

Atorvastatin is currently the most frequently used lipid-lowering drug in this group of Colombian patients, especially in monotherapy and at doses close to the defined daily dose. Only half received high-intensity doses. New studies are required to verify the efficacy of these therapies.

## Resumen

**Introducción.:**

Los fármacos hipolipemiantes, especialmente las estatinas, han demostrado gran relevancia para la prevención y el tratamiento de las enfermedades cardiovasculares.

**Objetivo.:**

Determinar los patrones de prescripción de los fármacos hipolipemiantes y las variables asociadas con su uso en una población de Colombia.

**Materiales y métodos.:**

Se trata de un estudio descriptivo y transversal. A partir de una base de datos de dispensación de medicamentos de 4,5 millones de afiliados al sistema de salud de Colombia, se identificaron los pacientes de cualquier edad y sexo en tratamiento con hipolipemiantes (estatinas, fibratos, ezetimibe), entre enero y marzo de 2017. Se incluyeron variables demográficas, farmacológicas y de comedicaciones.

**Resultados.:**

Se identificaron 103.624 pacientes en tratamiento con hipolipemiantes. La edad promedio fue de 67,5 años y el 49,8 % tenía 65 o más años. El 58,0 % eran mujeres. Las estatinas fueron los más utilizados (n=96.910; 93,5 %), siendo la atorvastatina (n=80.812; 78,0 %) y la lovastatina (n=12.621; 12,2 %) las más frecuentes. La dosis promedio de atorvastatina fue de 30,3 mg/día y el 49,9 % de sus usuarios recibía presentaciones de 40 mg o más. Un total de 9.258 (8,9 %) pacientes recibían fibratos y solo 780 (0,8 %) tomaban ezetimibe. El 94,9 % de casos recibió tratamiento en monoterapia hipolipemiante y el 97,3 % (n=100.813) tenía comedicaciones para comorbilidades, siendo las más frecuentes antihipertensivos (89,1 %), antiagregantes plaquetarios (57,8 %), antidiabéticos (31,5 %) y antiulcerosos (34,2 %).

**Conclusiones.:**

La atorvastatina es actualmente el medicamento hipolipemiante más utilizado en este grupo de pacientes de Colombia, especialmente en monoterapia y a dosis cercanas a las definidas, aunque solo la mitad recibían dosis recibían dosis de alta intensidad. Se requieren nuevos estudios que verifiquen la efectividad de estos tratamientos.

Cardiovascular disease is a primary cause of morbidity and mortality worldwide, and its incidence continues to rise due to aging populations and unhealthy lifestyles ^1^. Colombia is not exempt to this situation. From 2005 to 2014, cardiovascular disease accounted for up to 30% of the country’s total deaths ^2^.

One of the primary risk factors associated with cardiovascular disease is dyslipidemia, which mainly includes altered total cholesterol levels and low-density lipoprotein (LDL) ^3^. Among the drugs useful for managing dyslipidemias are statins, fibrates and ezetimibe. Despite its effects on plasma lipid levels, the use of some of these pharmacological groups is lower than expected, even in high cardiocerebrovascular-risk populations ^4^^,^^5^.

The last study on usage patterns for this drug group in Colombia was published in 2008, where the only statin identified was lovastatin, and gemfibrozil was the second most used drug, reaching 27% ^6^. More molecules are now available in all lipid-lowering drug groups, and reports in Colombian populations show some changes in their prescriptions. For example, by the year 2013, atorvastatin had already displaced lovastatin as the most widely used statin drug ^7^^,^^8^.

In the present work, we sought to determine the prescription patterns of lipid-lowering drugs in patients affiliated with the *Sistema General de Seguridad Social en Salud* (SGSSS) in 2017, considering the posible epidemiological changes in cardiovascular disease and the inclusion of new lipid-lowering medications in the benefit plan offered by health insurance entities (Benefit Plan Administrators) ^9^.

## Materials and methods

### Study design

A cross-sectional study was conducted on the prescription habits of lipid-lowering drugs. Information was obtained from a population database of approximately 4.5 million people affiliated with the SGSSS in five Benefit Plan Administrators of the contributory scheme, which corresponds to approximately 22.5% of the active population affiliated with this regime in the country and 9.3% of the Colombian population.

We analyzed prescribing data from patients treated with lipid-lowering drugs between January 1st. and March 31st., 2017 in all Colombian cities with reliable databases. We included data on individuals of all ages and sexes who had been prescribed lipid-lowering drugs and whose management was uninterrupted for at least three months to ensure that patients stably complied with the treatment, reflecting medication tolerance and adherence.

Medicine consumption information on the affiliated population was systematically obtained by the dispensing company (Audifarma, S. A.). Audifarma, S. A. is the largest drug dispensing company in Colombia. Information is generated at the time of dispensing and stored on a server with daily dispensing data (approximately 2.8 million formulas per month). To access the data, the Business Object tool is used on a platform in Oracle where all the drug claims are stored; and a database was designed with the following variables:

1. Sociodemographics included sex, age, and city.
2. Lipid-lowering drugs included statins (atorvastatin, cerivastatin, fluvastatin, lovastatin, pitavastatin, pravastatin, rosuvastatin, simvastatin), fibrates (bezafibrate, ciprofibrate, fenofibrate, gemfibrozil) and ezetimibe (alone or combined with statins). The dose and quantity delivered were recorded. The defined daily dose (DDD) was used as a unit of measurement for the drug use per the recommendations of the World Health Organization.
3. Co-medication was accepted as a surrogate indicator of chronic disease, considering the following circumstances: a) antidiabetics and insulins/diabetes mellitus; b) antihypertensive and diuretic/arterial hypertension; c) anticoagulants/thromboembolic disorders; d) levothyroxine-antithyroid/thyroid pathology; e) nitrovasodilators/ischemic heart disease; f) anti-ulcers/acid-peptic disease; g) antiplatelet drugs/cardiovascular prevention.

### Bioethics

The protocol was endorsed by the Bioethics Committee of the *Universidad Tecnológica de Pereira* and was classified in the category of “risk-free research”. Patients’ personal data were not used, and their identities were safeguarded per the recommendations of the Declaration of Helsinki.

### Data analyses

Descriptive statistics were used to analyze the data using the statistical package SPSS™, version 24.0 (IBM, USA) for Windows. Bivariate tests were also performed, such as Student’s t test to compare quantitative variables, and c*2* for categorical variables. We prepared binary logistic regression models using lipid-lowering drug use alone or in combination therapy (yes/no) and the need for co-medication (yes/no) as dependent variables. The covariates in these models included age, sex and the variables that were significantly associated with the dependent variables in the bivariate analyses. The statistical significance level was p<0.05.

## Results

**Figure 1.**
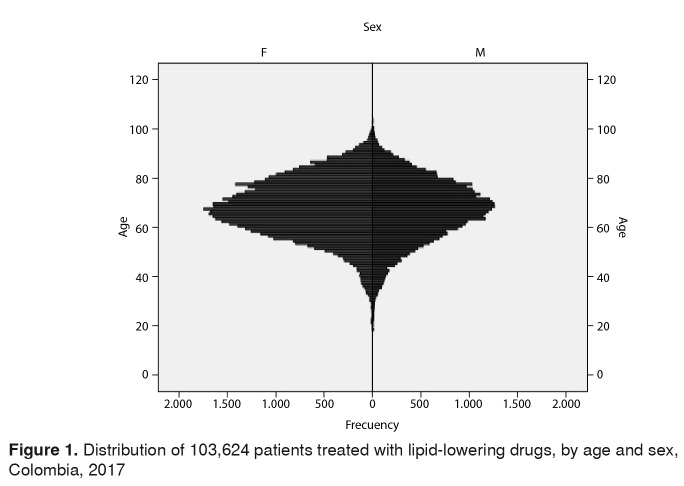
Distribution of 103,624 patients treated with lipid-lowering drugs, by age and sex, Colombia, 2017

We identified 103,624 patients affiliated with the SGSSS being treated with lipid-lowering drugs continuously during the study period. The average age was 67.5 ± 12.1 years (range: 18-105 years), and the sex distribution showed that 60,098 (58.0%) were women. People aged 65 years and older represented 49.8% (n=51,615) of the population (figure 1).

Of the population, 93.5% (n=96,910) received treatment with some statin, with atorvastatin being the most used (n=80,812; 78.0% of the total population), followed by lovastatin with 12,621 patients (12.2%). The main presentations dispensed were atorvastatin tablets 20 mg (n=42,389; 52.4% of those with atorvastatin) and 40 mg (n=39,724; 49.2%), but 3,210 (4.0%) received 10 mg, and 634 received 80 mg (0.8%). All lovastatin was prescribed as 20 mg. Only 4 patients received pitavastatin, and no formulations of cerivastatin, fluvastatin and pravastatin were found.

A total of 9,258 (8.9%) patients received fibrates, mainly gemfibrozil (n=8,242; 8.0% of the total subjects). Seven hundred eighty patients (0.8%) used some form of ezetimibe. Table 1 shows the prescription patterns of each of these drug groups.

**Table 1:** Prescription patterns of lipid-lowering drugs used in 103,624 patients in Colombia, 2017

Medication	Prescription/users				Dose (mg/day)		DDD	Female:Male	Mean age
									ratio		(years)
	Patients		%		Mean	Median					
				
				
									
Statins*	96,910		93.5					1.4:1	67.8
Atorvastatin	80,812		78.0	30.3		26.7	1.52	1.4:1	67.8
Lovastatin	12,621		12.2	19.9		20.0	0.44	1.7:1	68.2
Rosuvastatin	5,609		5.4	25.3		18.7	2.53	1.3:1	65.6
Simvastatin	343		0.3	29.3		24.9	0.98	1.1:1	67.2
Fibrates	9,258		8.9					1.0:1	63.2
Gemfibrozil	8,242		8.0	628.6		600.0	0.52	1.1:1	63.5
Fenofibrate	591		0.6	186.4		200.0	0.93	0.8:1	61.3
Ciprofibrate	471		0.5	97.5		100.0	0.97	0.7:1	60.6
Ezetimibe	780		0.8	9.0		9.3	0.90	0.9:1	65.3
Ezetimibe 10 mg	262		0.3	8.1		6.7	0.67	1.0:1	65.0
Combined presentations	524		0.5	9.3		9.3	0.93	0.9:1	65.6

DDD: average ratio between the prescribed daily dose and the defined daily dose.

* Only 4 patients were receiving pitavastatin during the study time.

The relationship between the average dose administered and the DDD varied greatly, with values from 0.44 for lovastatin to 2.53 for rosuvastatin (table 1). A total of 40,358 patients with atorvastatin (38.9% of the total population and 49.9% of those with atorvastatin) were receiving presentations of 40 mg or more, while 5,274 patients received rosuvastatin 20 mg or more (5.1% of all patients and 94.0% of rosuvastatin users).

### Monotherapy versus combination therapy

**Figure 2.**
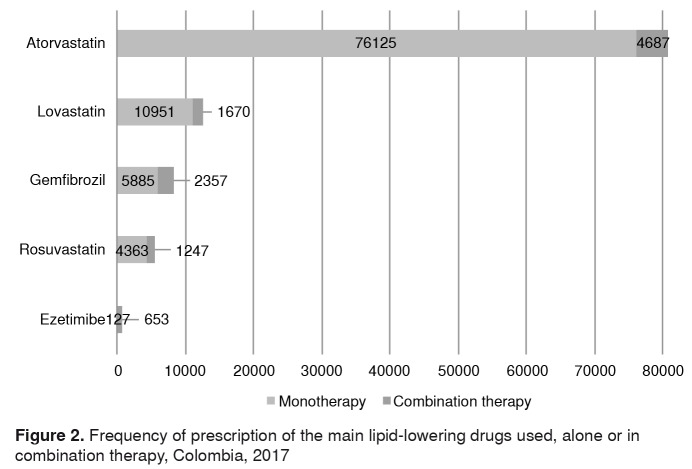
Frequency of prescription of the main lipid-lowering drugs used, alone or in combination therapy, Colombia, 2017

A total of 98,355 patients (94.9%) used a single lipid-lowering drug during the study period, while 5,190 (5.0%) used a combination of two drugs, and 79 cases received three medications. Figure 2 shows the distribution of the main lipid-lowering agents used by monotherapy or combination therapy.

The most frequent association was atorvastatin plus gemfibrozil (n=1,986 patients, 1.9% of all patients). Multivariate analysis of the relationship between the use of combination lipid-lowering therapy and other variables showed that the concomitant use of antidiabetics, thiazide diuretics, minoxidil and proton pump inhibitors increased the probability of receiving therapy with more than one lipid-lowering drug. In contrast, being 65 years of age or older and using apixaban reduced this probability (table 2).

**Table 2:** Variables associated with combined lipid-lowering therapy in binary logistic regression models, Colombia, 2017

Variables	Sig.	OR	95% CI	
		
Lower	Upper		
		
					
Age ≥65 years	<0.01	0.75	0.70	0.80	
Male sex	0.10	1.05	0.99	1.12	
Use of:					
Antidiabetics	<0.01	1.12	1.05	1.20	
Thiazide diuretics	<0.01	1.18	1.10	1.27	
Alpha-blockers	0.09	0.87	0.74	1.02	
Minoxidil	0.04	2.41	1.03	5.68	
Apixaban	0.04	0.48	0.24	0.96	
Proton pump inhibitors	<0.01	1.16	1.09	1.24	
Living in Cali city	<0.01	1.43	1.30	1.57	
Constant	0.00	0.05			

Sig.: Significance; OR: Odds ratio; 95 % CI: 95 % confidence Interval

### Co-medication

Most study patients (97.3%, n=100,813) used some co-medication. The average number of co-medications per patient was 4.0 ± 2.0 (range: 0-14 different drugs). The drugs for other comorbidities most frequently identified were antihypertensive drugs (n=92,338; 89.1%), antiplatelet agents (n=59,890; 57.8%, and 97.8% corresponded to acetylsalicylic acid), antidiabetics (n=32,609; 31.5%, of which 30.4% used insulins) and antiulcer drugs (n=35,397; 34.2%, where 92.2% corresponded to proton pump inhibitors).

We identified 28,562 patients (27.6%) consuming antihypertensive and antidiabetic medications concomitantly. Of diabetic patients, 87.6% received an antihypertensive, while 30.9% of hypertensive patients used antidiabetics.

In the antihypertensive group, 41,510 patients (40.1% of the total patients, 45.0% of those with antihypertensive drugs) received a combination of two drugs in this group, and 17,589 (17.0%) received three. The most prescribed group was angiotensin II-receptor antagonists (especially losartan) in 65,416 patients (63.1%), followed by β-adrenergic blockers (33.3%), calcium channel blockers (32.9%), thiazide diuretics (23.3%) and angiotensin-converting enzyme inhibitors (17.2%).

Other medications included those used to treat thyroid disease (n=18,242, 17.6%), loop diuretics (n=15,063, 14.5%), aldosterone inhibitors (n=5,501, 5.3%), anticoagulants (n=3,405, 3.3%), nitrovasodilators (n=1,326, 1.3%) and arterial vasodilators (n=73, 0.1%). Table 3 shows the sociodemographic and pharmacological variables of patients receiving lipid-lowering drugs by comedications or comorbidities.

**Table 3:** Comparison of lipid-lowering prescriptions according to comorbidity, Colombia, 2017

	Anti			Antiplatelet		Antidiabetics			Antiulcer			Thyroid		Loop		Aldosterone			Anticoagulants	
Variable	hypertensive			drugs					drugs			hormone		diuretic		inhibitors				
								
	n=92,338			n=59,890			n=32,609		n=35,397			n=18,242		n=15,063		n=5,501			n=3,405	
Mean age (years)	68.1		68.8				67.3		69.5		69.5		73.4		70.1			71.4	
Women (%)	53,480	(57.9)	32,478			(54.2)			18,290	(56.1)	22,753	(64.3)	13,197		(72.3)	8,619		(57.2)	2,692		(48.9)	1,650	(48.5)
Polytherapy (%)	4,659	(5.0)	3,068			(5.1)			1,793	(5.5)	1,924	(5.4)	969		(5.3)	742		(4.9)	272		(4.9)	153	(4.5)
Prescription- n (%)																			
Statins	87,167	(94.4)	56,878			(95.0)			30,477	(93.5)	33,515	(94.7)	17,305		(94.9)	14,432		(95.8)	5,391		(98.0)	3,344	(98.2)
Atorvastatin	72,875	(78.9)	48,175			(80.4)			25,739	(78.9)	28,547	(80.6)	14,475		(79.3)	12,464		(82.7)	4,757		(86.5)	2,915	(85.6)
Lovastatin	11,406	(12.4)	6,841 (11.4)				3,484	(10.7)	3,632	(10.3)	2,316	(12.7)	1,548	(10.3)	374	(6.8)		262	(7.7)
Rosuvastatin	4,904	(5.3)	3,222			(5.4)			1,939	(5.9)	2,159	(6.1)	910		(5.0)	741		(4.9)	396		(7.2)	252	(7.4)
Simvastatin	234	(0.3)	152			(0.3)			99	(0.3)	97	(0.3)	78		(0.4)	34		(0.2)	25		(0.5)	15	(0.4)
Fibrates	7,437	(8.1)	4,475			(7.5)			3,081	(9.4)	2,830	(8.0)	1,407		(7.7)	1,006		(6.7)	205		(3.7)	102	(3.0)
Gemfibrozil	6,891	(7.5)	4,136			(6.9)			2,729	(8.4)	2,571	(7.3)	1,266		(6.9)	924		(6.1)	168		(3.1)	76	(2.2)
Fenofibrate	327	(0.4)	196			(0.3)			222	(0.7)	153	(0.4)	73		(0.4)	48		(0.3)	20		(0.4)	18	(0.5)
Ciprofibrate	254	(0.3)	164			(0.3)			154	(0.5)	122	(0.3)	75		(0.4)	42		(0.3)	18		(0.3)	9	(0.3)
Ezetimibe	494	(0.5)	339			(0.6)			211	(0.6)	196	(0.6)	132		(0.7)	62		(0.4)	51		(0.9)	34	(1.0)
Ezetimibe 10mg	156	(0.2)	113			(0.2)			58	(0.2)	62	(0.2)	26		(0.1)	18		(0.1)	19		(0.3)	12	(0.4)
Combined presentations	339	(0.4)	227			(0.4)			154	(0.5)	135	( 0.4)	106		(0.6)	45		(0.3)	32		(0.6)	22	(0.6)

Multivariate analysis of the outcome of receiving some co-medication in patients being treated with lipid-lowering drugs indicated that being 65 years of age or older and using non-combined presentations of lovastatin, atorvastatin, rosuvastatin and fibrates were associated with a higher probability of receiving co-medication. Ezetimibe use was associated with a lower probability of receiving co-medication (table 4).

**Table 4:** Variables associated with lipid-lowering therapy with co-medication in binary logistic regression models, Colombia, 2017

										
Variables							Sig.	OR			95% CI		
											Lower		Upper	
											
							
Age ≥65 years				<0.01			3.54		3.22				3.88	
Male sex					0.22	1.05			0.97				1.15	
Use of:											
	Lovastatin				<0.01		6.70		5.22				8.60	
	Atorvastatin				<0.01		8.53		6.86				10.60	
	Simvastatin						0.52	0.76			0.33			1.77	
	Rosuvastatin				<0.01		3.32		2.60				4.24	
	Ezetimibe				<0.01		0.24		0.17				0.33	
	Fibrates				<0.01		1.64		1.32				2.04	
Constant					0.00	3.32								

Sig: Significance; OR: Odds ratio; 95 % CI: 95 % confidence interval

### Comparison between cities

Demographic variables and some prescription indicators were compared among the 105 Colombian cities included in this study. The different characteristics and proportions of lipid-lowering drug use in the eight main cities are summarized in table 5.

**Table 5:** Comparison of demographic variables and indicators of lipid-lowering prescriptions in eight Colombian cities, 2017

	Bogotá			Pereira		Cali		Cartagena		Barranquilla			Manizales			Ibagué		Palmira		Colombia
	n=32,670			n=9,378		n=8,969		n=6,022		n=5,520			n=4,155			n=3,488		n=3,174		n=103,624
Mean age (years)	64.8		68.8		71.9		66.1		67.8		66.6		67.0		70.8		67.5	
Women (%)	18,921	(57.9)	5,504	(58.7)	5,389	(60.1)	3,370	(56.0)	3,126	(56.6)	2,512	(60.5)	2,081	(59.7)	1,933	(60.9)	60,098	(58.0)
Polytherapy (%)	1,570	(4.8)	348	(3.7)	593	(6.6)	260	(4.3)	285	(5.2)	165	(4.0)	163	(4.7)	202	(6.4)	5,269	(5.1)
Comedication (%)	31,403	(96.1)	9176	(97.8)	8,683	(96.8)	5,934	(98.5)	5,449	(98.7)	4,023	(96.8)	3,428	(98.3)	3,119	(98.3)	100,813	(97.3)
Prescription - n (%)																		
Statins	30,783	(94.2)	9,000	(96.0)	8,104	(90.4)	5,795	(96.2)	5,313	(96.3)	3,973	(95.6)	3,214	(92.1)	2,795	(88.1)	96,910	(93.5)
Atorvastatin	25,131	(76.9)	7,290	(77.7)	7,136	(79.6)	4,677	(77.7)	4,596	(83.3)	2,811	(67.7)	2,618	(75.1)	2,274	(71.6)	80,812	(78.0)
Lovastatin	4,774	(14.6)	1,375	(14.7)	1,019 (11.4)		289	(4.8)	259	(4.7)	1,079	(26.0)	426	(12.2)	497	(15.7)	1,2621	(12.2)
Rosuvastatin	1,571	(4.8)	506	(5.4)	105	(1.2)	982	(16.3)	604	(10.9)	169	(4.1)	241	(6.9)	92	(2.9)	5,609	(5.4)
Simvastatin	171	(0.5)	25	(0.3)	37	(0.4)	9	(0.1)	10	(0.2)	2	(0.0)	3	(0.1)	13	(0.4)	343	(0.3)
Fibrates	2,549	(7.8)	507	(5.4)	1,199	(13.4)	318	(5.3)	332	(6.0)	249	(6.0)	359	(10.3)	480	(15.1)	9,258	(8.9)
Gemfibrozil	2,269	(6.9)	433	(4.6)	1,083	(12.1)	295	(4.9)	300	(5.4)	200	(4.8)	341	(9.8)	380	(12.0)	8,242	(8.0)
Fenofibrate	148	(0.5)	55	(0.6)	67	(0.7)	17	(0.3)	15	(0.3)	28	(0.7)	4	(0.1)	77	(2.4)	591	(0.6)
Ciprofibrate	138	(0.4)	22	(0.2)	50	(0.6)	7	(0.1)	19	(0.3)	22	(0.5)	17	(0.5)	31	(1.0)	471	(0.5)
Ezetimibe	314	(1.0)	49	(0.5)	124	(1.4)	22	(0.4)	14	(0.3)	18	(0.4)	3	(0.1)	44	(1.4)	780	(0.8)
Ezetimibe 10mg	57	(0.2)	23	(0.2)	72	(0.8)	8	(0.1)	2	(0.0)	9	(0.2)	1	(0.0)	13	(0.4)	262	(0.3)
Combined presentations	257	(0.8)	26	(0.3)	56	(0.6)	14	(0.2)	12	(0.2)	9	(0.2)	2	(0.1)	31	(1.0)	524	(0.5)

## Discussion

In the present work, the utilization profile of the main lipid-lowering drugs used in an insured population of the Colombian health system was described, finding large differences from data previously reported in the country.

The average age of those who use these medications is consistent with the profile of dyslipidemia and cardiovascular disease ^2^^,^^10^. The predominance of females attracts attention because men experience greater mortality and more years of life potentially lost due to this group of pathologies ^2^, although in Colombia, mixed dyslipidemia has been described as the most common and that it presents more frequently in women ^10^.

During the study period, the most used drug group was the statins, encompassing over 90% of the total prescriptions, while fibrates only accounted for approximately 9%. This shows an important change regarding the 2008 data, where statins, despite also being the most consumed, only encompassed 71% of total prescriptions and fibrates comprised 27% ^6^. As mentioned above, for 2008, the dominant statin was lovastatin because it was the only one included on the SGSSS benefit plan’s drug list. The change in the prescription pattern to atorvastatin, until now the dominant drug, was likely due to its inclusion in the benefit plan ^9^ and because higher doses can be achieved, which improves the therapy’s intensity and therefore the expected efficacy ^11^^-^^13^.

This change towards increased atorvastatin prescriptions is also supported by previous national study results, which found that patients treated with lovastatin were less likely to achieve metabolic control (although it was used at doses lower than those recommended, similar to the present study) ^8^. In addition, atorvastatin has been identified as the most cost-effective option for patients with dyslipidemia in the Colombian health context when moderate to high intensity treatments are required ^14^. When comparing these results with studies from other countries, atorvastatin has also been the most prescribed in Taiwan, but only at 37% ^15^, whereas research in Italy and Brazil found a predominance of simvastatin ^16^^,^^17^.

In the present study, more than a third of the population was receiving statins at high intensity doses by international standards (atorvastatin 40-80 mg, rosuvastatin 20-40 mg) ^12^. In a sample of Colombian patients receiving lipid-lowering drugs, according to the Framingham scale, 94% of these patients had a moderate to high cardiovascular risk ^10^, which is why it would be expected that more patients in the study were receiving high doses of these drugs in addition to the indications of national and international management guidelines ^12^^,^^13^.

Lipid-lowering drug use in combination therapy was 5%, which is similar to previous national reports ^8^ and works published in France ^18^ and the United States ^19^. Concomitantly using statins with fibrates is usually avoided due to the increased risk of adverse reactions, which has been documented in pharmacoepidemiological studies ^19^; therefore, the high monotherapy frequency seems appropriate, although in adequately selected patients with residual cardiovascular risk, combination therapy may be a therapeutic option of interest to achieve goals ^20^ with fibrates as well as ezetimibe ^21^, and more recently with new therapeutic options such as PCSK9 inhibitors ^3^.

The comorbidities and co-medications found in the study population are consistent with the profile associated with patients requiring lipid-lowering drugs. Therefore, antihypertensive, antiplatelet and antidiabetic agents were the dominant co-medications, as has been described in other studies ^8^. It is striking that in this patient group, antiplatelet agents reached a usage near 60%, much higher than the value of 3.8% reported in 2008, indicating an improvement in preventing cardiovascular risk among this type of patients ^6^.

The worldwide tendency is to increase prescriptions of lipid-lowering drugs in patients with diabetes mellitus ^22^, because the recommended use of statins is growing in patients with this comorbidity as a routine step for prevention of cardiovascular events, which was also identified in this study (increasing from 20% in 2008 to 32% in 2017) ^6^. The high use of antiulcerative drugs has been described previously, especially in chronic disease populations, often without a clear indication ^23^, although in this case, it can be explained by the high prescription of antiplatelet agents in elderly patients.

Another interesting aspect is the relationship found between ezetimibe use and the lower probability of having co-medications, especially because ezetimibe has been described as a useful medication to achieve goals in patients with dyslipidemia who also have multiple comorbidities ^24^. Residual confounders not considered during the study may explain this relationship, such as the patient’s educational levels or socioeconomic statuses ^25^, aspects of health and insurance services, preferences of prescribing physicians or the presence of clinical inertia to reach goals in dyslipidemia in patients with multiple pathologies ^26^.

Differences between prescription profiles in different cities (for example, variations in the prevalence of rosuvastatin use) are frequent in pharmacoepidemiological studies ^6^ and have been associated with local differences in health services, medical training or inhabitants’ characteristics ^27^.

This research has some limitations. Because the results came from dispensing databases, and clinical records were not consulted, variables such as patients’ cardiovascular risk, lipid profiles, efficacy or the presence of adverse reactions were not analyzed. Likewise, the patterns found are applicable only to populations with insurance characteristics similar to the study subjects. New research is required to determine detailed aspects of using these drugs, for example, by the indication of treatment in the field of primary and secondary cardiovascular disease prevention.

The prescription patterns identified in this study highlight that the main lipid-lowering drug dispensed in Colombia is atorvastatin, especially in monotherapy and at doses close to DDD.

Regarding previous analyses, the prescriptions differed considerably, likely due to modifications in the drugs included in the benefit plans and the evidence presented in different clinical guides, highlighting the increase in useful co-medications for cardiovascular prevention such as antiplatelet drugs.

These data may be useful for professionals and health administrators to define behaviors related to managing cardiovascular risk in the Colombian population, since there seems to be an opportunity for improvement related to a probable underuse of statins at high doses in patients who require them. The adequate management of dyslipidemias can have a long-term impact on public health by reducing cardiovascular risk, morbidity and mortality secondary to ischemic events, heart failure and, in addition, the costs derived from their attention.
